# Antimicrobial Stewardship Programs in Pediatric Intensive Care Units: A Systematic Scoping Review

**DOI:** 10.3390/antibiotics14020130

**Published:** 2025-01-26

**Authors:** Cecilia Liberati, Giulia Brigadoi, Elisa Barbieri, Carlo Giaquinto, Daniele Donà

**Affiliations:** Division of Paediatric Infectious Diseases, Department of Women’s and Children’s Health, University of Padova, 35128 Padua, Italy

**Keywords:** antimicrobial resistance, antimicrobial stewardship, diagnostic stewardship, pediatric intensive care unit

## Abstract

**Objectives**: We aimed to summarize the current state of antimicrobial stewardship (ASP) and diagnostic stewardship programs (DSPs) implemented in pediatric intensive care units (PICUs). **Methods**: Embase, MEDLINE, Scopus and the Cochrane Library were searched, including studies from 1 January 2007 to 20 February 2024. Studies were included in the review if they assessed the implementation of an ASP or a DSP in a PICU. Identified references were downloaded into Rayyan software, and data were extracted using a standardized data collection form. **Results**: 18 studies were included; 13 described an ASP intervention, and 5 described a diagnostic stewardship intervention. Most studies were retrospective and adopted a persuasive strategy for ASP, reporting positive effects on antimicrobial consumption. However, studies were dramatically heterogeneous in terms of intervention type, outcomes and metrics used, limiting the possibility of a broader comparison. Diagnostic stewardship studies included mainly the impact of biomarkers and pathogen testing panels without significant impact on antibiotic prescription patterns. Antimicrobial resistance changes were not described by the majority of studies. **Conclusions**: the implementation of ASP in PICUs is still limited, with significant variability in the metrics used to evaluate outcomes. To enhance the effectiveness of these programs, it is crucial to harmonize reporting metrics to allow an adequate comparison of results and to find the best strategies to inform ASP in PICUs.

## 1. Introduction

Antimicrobial resistance (AMR) has been identified by the World Health Organization as one of the ten global health threats [1]. In 2019, it was estimated that 4.95 million deaths worldwide were associated with bacterial AMR, with 1.27 million deaths directly attributable to it [2]. Although commonly perceived as an issue affecting primarily adults and the elderly, a 2019 study in Europe on the attributable deaths and disability-adjusted life-years caused by infections with antibiotic-resistant bacteria in the European Union revealed that the population most at risk was infants under one year of age [3,4]. Furthermore, pediatric patients face a shortage of new antibiotics effective against multidrug-resistant organisms (MDROs), as many of these drugs are used off-label in children with limited experience and insufficient knowledge regarding proper dosing, pharmacokinetics and pharmacodynamics, particularly in children with comorbidities.

Patients at higher risk of infections caused by MDROs are often those with chronic conditions or those admitted to pediatric intensive care units (PICUs). Children in PICUs are typically more fragile, suffering from life-threatening illnesses, frequently with underlying chronic conditions [5,6]. In this context, distinguishing between infectious diseases and other inflammatory conditions can be particularly challenging, as their clinical presentations may overlap, and laboratory tests may not always provide clear differentiation. Inflammatory markers, such as C-reactive protein (CRP) and procalcitonin, can be elevated in non-infectious conditions like inflammatory diseases or renal insufficiency [7,8]. Additionally, microbiological test results are not immediate, often requiring several hours or even days to be fully processed, leaving physicians to make empirical treatment decisions. This situation frequently leads to higher use of antibiotics, particularly broad-spectrum ones, compared to general wards, with challenges in de-escalating or discontinuing antibiotic therapy. Over time, this indiscriminate use of antibiotics has contributed to the rise of antibiotic resistance [9].

To contain the spread of resistant organisms, antimicrobial stewardship programs (ASPs) should prioritize education on preventing hospital-acquired infections and optimizing antimicrobial use [10]. While ASPs are encouraged across Europe, a recent survey indicates that less than 60% of PICUs implement regular rounds and audits for these programs [11].

ASPs have demonstrated effectiveness in various settings, both inpatient and outpatient, for reducing antibiotic consumption among children and adults [12,13]. However, given the unique challenges in PICUs and the vulnerability of the children admitted, specific interventions are needed that foster communication and trust-based relationships between prescribers and ASP team members.

Our systematic scoping review aims to summarize the current state of antimicrobial stewardship and diagnostic stewardship programs (DSPs), with a focus only on the use of biomarkers and microbiological results to improve antibiotic prescription implemented in PICUs worldwide, thereby informing best practices in this critical area.

## 2. Results

**Figure 1:**
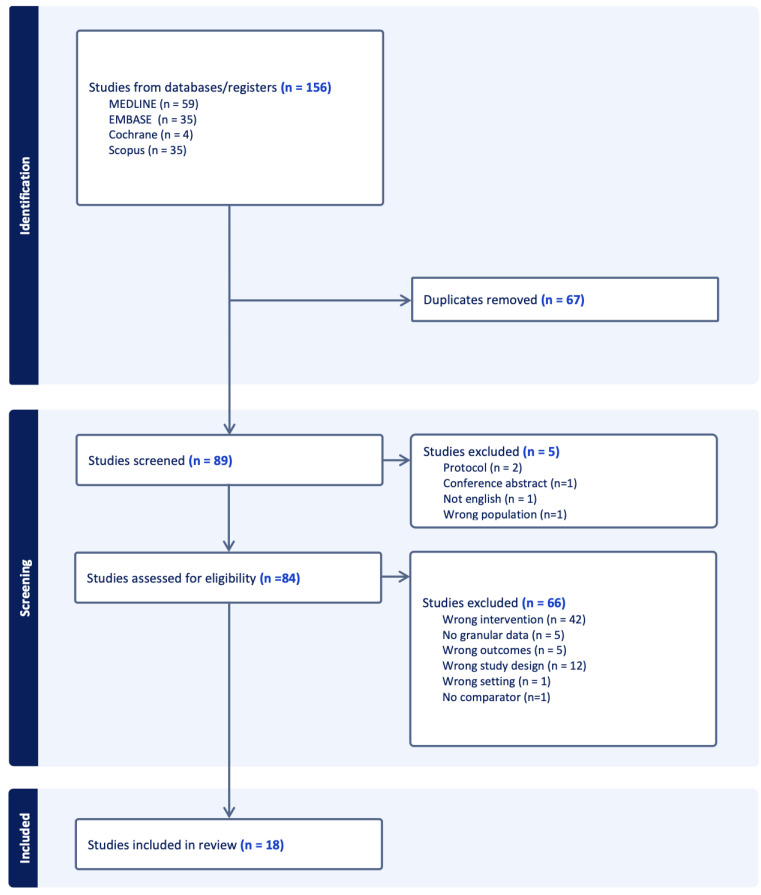
PRISMA flowchart of included studies.

A total of 18 studies were included in this scoping review from the 156 references obtained through the search of three different databases (Figure 1). The characteristics of the included studies are reported in Table 1 (ASP) and Table 2 (DSP).

Most studies were conducted in High-Income Countries (14 out of 18). Thirteen studies described formal antimicrobial interventions in PICUs [14,15,16,17,18,19,20,21,22,23,24,25,26], whereas five studies regard diagnostic stewardship approaches [27,28,29,30,31]. Four studies of this latter group focused on selected populations in PICU: patients with systemic inflammatory response syndrome (SIRS) or patients with respiratory infections [27,29,30,31]. For the ASP interventions, nine studies considered all patients in the PICU [14,16,17,18,19,20,22,25,26], one study was conducted in a cardiothoracic PICU [15], two studies focused on patients with respiratory infections [21,24] and one study on patients with healthcare-associated infections [23].

**Table 1:** Characteristics of the studies regarding Antimicrobial Stewardship Program included in the systematic scoping review.

Author, Year, Setting	Study Design	Population	Intervention	Outcome	Key Findings
Audit and Feedback, presence of PID at ward round or positive Feedback					
Haque et al., 2018 [15], LMIC	Retrospective study comparing pre-intervention (Jan–Mar 2016) with post-intervention period (Apr–Jun 2016). Short report	Cardiothoracic PICU	Prospective audit and feedback	Antibiotic consumption by measuring total DOT/1000 pd in the pre- and post-intervention periods.	There was a 64% reduction in antibiotic utilization in ASP period. The appropriate use of empirical antibiotic therapy for culture-negative infection-like symptoms increased from 6% to 45%
Aizawa et al., 2018 [16], HIC	Retrospective study between Apr 2010 and Dec 2015	All patients admitted	Pediatric infectious disease physicians attended the PICU morning rounds every day	The primary outcome was the consumption of antipseudomonal agents, as measured by DOT/1000 pd. ITS performed.	Significant reduction in the level and trend of DOT/1000 pd for total antipseudomonal agents (−24%); significant change in trend but not in level of DOT/1000 pd for non-antipseudomonal agents
Adams, 2019 [17], HIC	Retrospective cohort study, pre- and postimplementation quality improvement study (Jul 2015–Mar 2016)	All patients admitted	Mandatory antimicrobial time-out 48–72 h after initiation of therapy	Primary outcome: DOT/1000 pd for vancomycin, meropenem and piperacillin/tazobactam and DOT for all antibiotics	Overall significant reduction for DOT/1000 pd for overall antibiotics, meropenem and vancomycin
Jones et al., 2019 [18], HIC	Prospective non-randomized study, Apr 2017–Mar 2018	All patients admitted	Positive feedback for behaviors via reports and interviews	Antibiotic consumption measured by dispended doses/PICU bes-days	Overall reduction by 6.5%, meropenem reduction by 17.5%
Renk et al., 2020 [20], HIC	Prospective, pre- and post-implementation cohort study (Jan 2017–Jun 2017 and Jan 2018–Jun 2018)	All patients admitted	Weekly PID ward round, audit and feedback	Antibiotic consumption by DOT/1000 ppd and LOT/1000 pd, PICU LOS, mortality, costs	Significant reduction of DOT/1000 pd by 18%. LoT/1000 ppd decreased not significantly. Vancomycin significantly decreased
Aljassim et al., 2021 [21], HIC	Multicenter retrospective cohort study (2 PICUs), pre- and postimplementation quality improvement study (Jul 2015–Mar 2016 and Jul 2016–Mar 2017)	Patients with bronchiolitis	Audit and feedback implemented in 1 PICU out of 2	Proportion of antimicrobials discontinued 72 h after hospital admission; anti-microbial treatment duration; antimicrobial prescriptions within 48 h of hospital admission	ASP is associated with increased odds of discontinuing antimicrobials but not with antimicrobial duration or antimicrobial prescriptions
Kit-Anan et al., 2022 [22], LMIC	Historical control study (Jul 2017–Dec 2018 and Apr 2019–Sep 2020)	All patients admitted	“Handshake” approach ASP, no pre-authorization	Carbapenem consumption rate, measured by DOT/1000 pd	Carbapenem consumption significantly decreased
Alfraij et al., 2023 [25], HIC	Retrospective cohort study (Oct 2018–Oct 2020)	Admitted patients who received antimicrobials	Tele-ASP: weekly prospective audit and feedback by the ASP team, with PID specialist joining remotely	Antimicrobial consumption by DOT/1000 pd	A decline in DOT was observed across most antibiotic classes, except for ceftriaxone. In the analysis based on admission diagnosis, the decrease in antimicrobial consumption was significant only for cardiac diseases. No effect on the length of PICU stay, length of hospitalization or mortality was observed.
Zombori et al., 2023 [26], HIC	Multicenter retrospective study (Apr 19–Apr 21)	All patients admitted	The antimicrobial stewardship pre-dates the beginning of the study: twice weekly virtual handshake stewardship rounds on all ICUs discussing patients receiving antibiotics	Evaluation of antibiotic consumption by comparing the antimicrobial spectrum index (ASI) with DOTs	Median ASI/antibiotic days: Immunocompromised patients received much broader-spectrum antibiotics than immunocompetent patients. Patients who had stewardship input had a higher ASI compared with those who did not throughout the whole period. ASI shows less variability than DOT.
Preauthorization plus guidelines and education					
Wassef et al., 2020 [19], LMIC	Prospective study (Apr 2016, Jun 2017)	All patients admitted	Guidelines, education, antibiotics time-out, pre-authorization	Clinical outcome of patients, LOS, DOT, LOT	No difference in mortality, reduced LOT and LOS, decrease in ceftriaxone and amikacin consumption, increase in gentamicin, levofloxacin, clindamycin
Guidelines and education					
Lee et al., 2016 [14], HIC	Retrospective chart review before and after intervention (Sep 2010–Aug 2011 and Sep 2012–Aug 2013)	Patients in pediatric, neonatal cardiac ICUs	Guidelines and education	Monthly change in overall antibiotic and broad-spectrum antibiotic prescriptions by DOT/1000 pd	The overall antibiotic days of therapy in PICU decreased by 21%, and targeted broad-spectrum antibiotic days of therapy decreased by 75% after guideline implementation
Fan et al., 2023 [24], HIC	Retrospective study (May 2016 to April 2020)	Patients admitted with severe bacterial pneumonia	Education, regular inspections on antibiotics use	Antimicrobial resistance rates, antimicrobial consumption by DDD/1000 pd, antibiotic consumption and clinical outcome	Reduced resistances for S.pneumoniae, S.aureus, K.pneumoniae, A.baumanii. Cephalosporins, carbapenems, macrolides, antifungal agents and linezolid showed a decreasing consumption trend
Other interventions					
Oliveira da Silva et al., 2022 [23], LMIC	Longitudinal study (2007–2018)	Patients with Healthcare Associated Infection (HAI)	Monitoring software that flags patients with antimicrobial “alert”	Antibiotic consumption measured by DOT/1000 pd and antimicrobial resistance	Decrease in total antibiotic consumption; decrease in some antimicrobial resistances (Enterobacterales, *S.aureus*, non-fermenting gram-negative bacilli)

ASI: antibiotic spectrum index; DDD: daily defined doses; DOTs: days of therapy; HIC: high-income countries; ICU: intensive care unit; LMIC; low-middle-income countries; LOS: length of stay; LOT; length of therapy; PID: pediatric infectious disease; pd: patient days. Defined daily dose (DDD)/1000 pd: Doses defined as the average in adults either purchased, dispensed or consumed/patient days in a time period × 1000. Days of therapy (DOT)/1000 pd: sum of days of antimicrobial therapies administered in the unit/patient days in a time period × 1000. Length of therapy (LOT)/1000 pd: duration of antimicrobial use/patient days in a time period × 1000. Antibiotic spectrum index (ASI)/antibiotic days: Sum of ASI score of each antibiotic prescribed × days of that antibiotic.

**Table 2:** Characteristic of the studies regarding diagnostic stewardship program included in the systematic scoping review.

Author, Year, Setting	Study Design	Population	Intervention	Outcome	Key Findings
Downes et al., 2017 [27], HIC	Prospective cohort study Jan 2012–Mar 2014	Children with SIRS and suspected infection	Biomarker panel daily for 72 h at initiation of antibiotics	“Excess” days of therapy (days after 48 h in patients in which bacterial infection is excluded)	The best combination of biomarkers to identify patients at low risk of bacterial infection was CRP ≤ 5.0 mg/dL plus SAA ≤ 15 µg/mL. Patients without bacterial infection received a mean of 3.8 excess days of therapy
Katz et al., 2021 [28], HIC	Single center, randomized prospective clinical trial (Feb 2018–Apr 2019)	Critically ill children admitted to an ICU setting and started on intravenous antibiotics	Intervention: PCT testing protocol; comparison: usual care arm	Median antibiotic DOTs per patient in the first 14 days after enrollment	No difference in antibiotic DOT between study arms
Wagner et al., 2021 [29], HIC	Retrospective review (Dec 2018–Aug 2019)	Admitted children 1 month-18 year with SIRS, at least 1 PCT level and one blood culture. Excluded if immunocompromised, antibiotics administered for <48 h or antibiotics initiated at an outside hospital	PCT monitoring protocol to help guide antibiotic decision-making	Adherence to the protocol	Full adherence was observed in 34%. Reasons for non-adherence were excess PCT monitoring (54.5%), antibiotic continuation (30.3%) or both (15.2%)
Yoshida et al., 2021 [30], HIC	Single-center, pre-/post-study (Dec 2017–Nov 2018 and Mar 2019–Feb 2020)	Consecutive children with respiratory infections	Multiplex polymerase chain reaction testing panel (17 viruses and 3 bacteria) with following recommendations	Primary outcome: pathogen identification rate during pre- and post-intervention. Secondary outcome: use and duration of antibiotics within 14 days of admission to the PICU, before and after the implementation of mPCR testing	The panel increased the proportion of pathogen identification. No differences were observed in use and duration of broad-spectrum antibiotics
Brotons et al., 2022 [31], HIC	Prospective cohort study (Dec 2015–Feb 2017)	Patients aged < 18 years with clinical diagnosis of acute low respiratory tract infection	Rapid panel test of respiratory viral and atypical bacteria	Primary outcome: panel diagnostic performance compared to standard, antimicrobial changes consequence of panel results, days of antimicrobial saved that could be attributable to panel test use	The panel increased diagnostic yield of routine diagnostic assays. The main achievements were suspension of oseltamivir and macrolide use with early panel results

CRP: C-reactive protein; DOTs: days of therapy; HIC: high-income countries; ICU: intensive care unit; PCT: procalcitonin; SAA: serum amyloid A; SIRS: systemic inflammatory response syndrome.

### 2.1. Type of Intervention

The most common intervention reported was audit and feedback 10/18, 55.6% [15,16,17,19,20,21,22,24,25,26], whereas other interventions were less frequently implemented, for example, education and guidelines [14], education and guidelines with audit and feedback [19,24], audit and positive feedback [18], pre-authorization [19] and alert created on pre-established criteria by an informatic software [23].

Diagnostic studies evaluated the impact of biomarkers [27,28,29] or pathogen testing panels [30,31].

### 2.2. Metrics Used for Outcome Evaluation

Most studies (8 out of 18, 44.4% [14,15,16,17,20,22,23,25]) used days of therapy (DOT)/1000 patient-days as an indicator of antibiotic consumption in the outcome evaluation. Other indicators used were doses dispensed to PICU/PICU bed-days [18], defined daily dose (DDD)/1000 patient-days [24], antibiotic spectrum index [26] and proportion of prescription or antibiotic discontinuations [29].

### 2.3. Outcomes

Nine studies analyzed the overall antibiotic use, with five deepening the analysis to specific antibiotic classes. One study analyzed only carbapenem consumption [22], and only two studies reported antifungal consumption in PICU [19,24]. Most studies reported a reduction in antibiotic use without compromising patient outcomes. In some cases, a reduction in antimicrobial resistance rate was observed after the introduction of an ASP. Few studies considered other outcomes such as length of stay (LOS) or mortality, without reporting significant results.

Regarding DSP, in many cases, the implementation of a DSP did not change the antibiotic prescription pattern.

## 3. Discussion

Studies of antimicrobial stewardship in PICUs were highly heterogeneous in populations, types of interventions, outcomes and metrics used for antibiotic consumption. This reflects the lack of guidelines and the difficulty in translating antimicrobial stewardship intervention performed in other settings (e.g., general wards) to such a complex setting. While there is a significant body of the literature regarding bundles and prevention of hospital-acquired infections in PICU, strategies to reduce antimicrobial consumption are widely under-reported [12,32,33].

Most studies implemented a persuasive stewardship strategy combining audit and feedback, utilizing various methods, timing and informatics tools. The intervention targets were also diverse, encompassing heterogeneous patient populations. Even within the same PICU, the intensity of the intervention may differ depending on the specific patient. While it may be more straightforward to manage patients with clearly defined conditions (e.g., bronchiolitis), in some cases, the diagnosis can be less clear, making it more challenging to adjust antibiotic therapy appropriately. In studies by Aljassim [21] and Fan [24], the ASP intervention targeted patients with respiratory infections, describing increased odds of antibiotic discontinuation in bronchiolitis and decreased antimicrobial consumption in patients with severe bacterial pneumonia, respectively. Other studies included all patients admitted or patients with antimicrobial prescriptions, mostly describing positive results after interventions. However, the great variability of outcome measures makes it difficult to compare stewardship impacts among studies. Standardized outcomes for stewardship purposes are strongly needed to provide stronger results despite study variability, aiming for a meta-analysis approach. DOTs/1000 patient-days have been suggested as the most accurate metric to assess antibiotic consumption in pediatrics [34]. Of note, only half of the studies in this review adopt this parameter, limiting a broader comparison of consumption and intervention effectiveness. In pediatrics, dispensed doses are not an accurate reflection of actual antimicrobial consumption, as vial usage does not correspond precisely to the doses administered. Similarly, metrics such as days of therapy or the number of prescriptions, when not adjusted for patient days, can be influenced by admission rates, which are highly variable. However, when daily electronic data monitoring is not feasible, collection of data regarding prescriptions in specific periods more than once per year might be a valid solution to evaluate the efficacy of an ASP to compare the same ward before and after intervention or PICUs in different hospitals [35].

Beyond admission rates, PICUs usually have an intrinsic variability in patients’ severity, which could justify peaks and falls in antibiotic consumption. When daily data collection is available, the interrupted time series (ITS) analysis could properly evaluate the success or failure of an ASP by describing the immediate level change and the trend of antibiotic consumption after an intervention rather than the description of the cumulative utilization before and after [36,37]. However, the ITS was performed only in the study by Aizawa [16]. Another strategy to limit variability in consumption estimations is the one adopted by Zombori et al., in which the “Antimicrobial Spectrum Index”(ASI) was used in four PICUs, providing a reliable indicator for broad-spectrum antimicrobial consumption, less variable than DOTs [26]. Only one study in the review tried to stratify the complexity of patients admitted by correlating the results of consumption with days of extracorporeal membrane oxygenation [16]. Broader severity indexes at the ward level have not been used in the study analyzed.

In the current review, we did not find any randomized controlled trial to evaluate the impact of an ASP in PICU. RCTs would allow valid findings but are difficult to implement and require multicenter connections and great human and economic resources to accomplish stewardship in more places at the same time.

The fight against AMR is one of the most important objectives of ASP. AMR changes have been described in only two studies: the study by Fan et al. on patients admitted to PICU with severe bacterial pneumonia in a four-year timeframe reported a reduction in surveillance samples of resistance patterns of *S. pneumomiae* (clindamycin, tetracycline), S. aureus (tetracycline), K. pneumoniae (amoxicillin/clavulanic acid and trimethoprim/sulfamethoxazole) and *A. baumanii* (cefotaxime, trimethoprim/sulfamethoxazole), after two years of ASP [24]. The study by Oliveira da Silva reports a reduction in resistance of Enterobacterales to cephalosporins, of non-fermenting gram-negative bacilli to aminoglycoside and of S. aureus to oxacillin in patients with healthcare-associated infections after six years of implementation of an informatic software providing alert to physicians [23]. To adequately evaluate the impact of an ASP intervention on AMR, however, it should be ensured preventive measures (precautions, isolations) in the PICU and sample screening policy remain the same throughout the study period, as those could significantly influence the spread of AMR independently from antibiotic consumption. In this view, prevention of acquired infections/colonizations and antimicrobial stewardship are two faces of the same coin. A recent meta-analysis showed that reducing antimicrobial duration may increase or decrease colonization by MDROs, dependent upon individual and bacterial characteristics, emphasizing the importance of colonization monitoring to inform antimicrobial stewardship in PICUs [38].

Diagnostic stewardship approaches in PICUs have not shown the same level of success in reducing antimicrobial use as ASPs. The studies included a focus on evaluating biomarkers, primarily procalcitonin, in patients with confirmed or suspected bacterial infections and utilizing respiratory virus panels for those with respiratory infections [27,28,29,30,31]. The study by Brotons et al. evaluated a panel test of respiratory virus and bacteria and found a reduction in days on antimicrobials for oseltamivir and macrolide, while other antibiotics did not differ [31]. The testing panel implemented in the study by Yoshida did not result in a different antibiotic consumption [30]. Similar results were reported in other studies conducted in different settings. For instance, in a study published by Baer et al. in 2013, children with lower respiratory tract infections (LRTI) were randomized into two treatment arms: one following a new algorithm incorporating procalcitonin (PCT) and the other receiving standard care. While PCT use did not alter the number of children treated with antibiotics, it significantly reduced the duration of therapy—from 6.3 to 4.5 days for LRTI and from 9.1 to 5.7 days for pneumonia [39]. Similarly, the use of rapid viral tests in pediatric emergency departments, such as influenza antigen tests, has shown potential to reduce antibiotic use and enhance appropriate oseltamivir prescriptions, as highlighted in a recent systematic review and meta-analysis [40]. Nevertheless, the adoption of these tests in this setting remains debated, particularly given the high costs associated with these devices.

The main pitfalls of diagnostic method implementation are the research of the optimal diagnostic test for a site-specific infection, the selection of patients to test or not to test for the risk of overtreating colonizing bacteria, and the correct modalities of collection to avoid contaminations [41]. Moreover, new diagnostic stewardship programs focusing specifically on the criteria for microbiological tests and the method of collection should be conducted. Recent studies have shown that blood and respiratory cultures in critically ill children can be safely reduced, avoiding microbiological cultures in patients without suspicion of sepsis [42,43,44,45]. In this view, the collaboration of clinicians with the microbiological laboratory emerges as fundamental to the implementation of diagnostic stewardship protocols.

## 4. Materials and Methods

### 4.1. Study Design

This systematic scoping review was conducted following the Preferred Reporting Items for Systematic Reviews and Meta-analysis (PRISMA) guidelines [46]. The study protocol was registered in the International Prospective Register for Systematic Reviews (PROSPERO) under registration number CRD42024577856.

### 4.2. Search Strategy and Data Extraction

Embase, MEDLINE, Scopus and the Cochrane Library were searched for relevant studies, combining terms for “pediatrics”, “stewardship” and “pediatric intensive care unit”. The search strategy was restricted to English articles and limited to the period from 1 January 2007 to 20 February 2024. The full search strategy is reported in the Supplementary Material.

Identified references were downloaded into Rayyan software [47] and were screened for title and abstracts by two independent reviewers (C.L., G.B.). Any references which did not meet the inclusion criteria were excluded. Full-text copies of the remaining references were obtained and independently examined in detail by two reviewers (C.L., G.B.) in order to determine whether they meet all the inclusion criteria. Any disagreement regarding study selection was resolved by discussion with a third reviewer (D.D.). Data were extracted using a standardized data collection form, capturing information on study characteristics (authors, year of publication, study design, country, sampling period), the population included (for example, hemato-oncological patients, cardiosurgical patients, children with congenital heart disease), the type of stewardship intervention implemented and the outcomes assessed, along with the specific metrics used to evaluate them.

### 4.3. Eligibility Criteria

Randomized controlled trials, controlled and uncontrolled before-and-after studies, controlled and uncontrolled interrupted time series, and cohort studies were included in the review if they assessed the implementation of an antibiotic stewardship program (ASP) or a diagnostic stewardship program (DSP) in a pediatric intensive care unit (PICU). No restrictions were applied regarding the type of PICU (e.g., general PICUs or cardiothoracic PICUs), but only studies on neonatal ICUs were excluded. Systematic and narrative reviews, case series, notes, letters, conference abstracts and opinion articles were excluded. Moreover, studies that included both pediatric and adult populations were excluded if it was not possible to extract the pediatric data. Similarly, studies involving various wards, including PICUs, were excluded if data specific to the PICU were not available.

### 4.4. Outcomes

The primary outcome was the impact of the ASP on antibiotic prescribing behaviors (by directly evaluating consumption or other surrogates, e.g., prescriptions and adherence to recommendations) after the intervention. Secondary outcomes considered were AMR, PICU length of stay, mortality and costs.

## 5. Conclusions

This review has several limitations. The implementation of ASP in PICUs is still limited, as highlighted by the few studies included in our scoping review. Most studies are retrospective, introducing various sources of bias with unclear methodologies, making it difficult to extrapolate definitive conclusions. Nonetheless, it highlights the need for PICU-specific guidelines for antimicrobial stewardship implementation and reporting. Moreover, although positive results in terms of antimicrobial consumption are reported in the studies included, there is a need to harmonize the stewardship reporting metrics to adequately compare results and find the best strategies to inform ASP in PICUs.

However, this high variability in interventions tells us that stewardship strategies need to be adapted according to local capability, PICUs’ complexity and laboratory availability. Rapid microbiology diagnostic tools and biomarker monitoring are steps toward the implementation of protocols in support of PICU prescribers, but larger studies to fully evaluate modalities of implementation as part of an antimicrobial stewardship intervention are needed. Future research on antimicrobial stewardship should prioritize prospective study designs in order to provide larger and stronger results, limiting the occurrence of bias and confounding factors. In this view, ASP and DSP programs should be integrated into national healthcare policies, funded and should aim at providing multidisciplinary approaches, including dedicated health informatics, to gather stronger information.

## Data Availability

The data included in this meta-analysis consist of previously published data from other authors and do not represent new data.

## Supplementary Materials

The following supporting information can be downloaded at: https://www.mdpi.com/article/10.3390/antibiotics14020130/s1, Supplementary S1: Search strategy.
