# Supplementary material for: Antimicrobial Stewardship Programs in Pediatric Intensive Care Units: A Systematic Scoping Review
Source: Antibiotics (Basel). 2025 Jan 26;14(2):130. doi: 10.3390/antibiotics14020130 (PMC11852047; doi:10.3390/antibiotics14020130)
Supplement: Supplementary file 1 [file antibiotics-14-00130-s001.zip › antibiotics-3410095-supplementary.pdf]

Supplementary materials:

S1, Search strategy

1. (infan* or toddler* or pre-schooler* or preschooler* or child* OR children or adolescen* or pediatr* or paediatr* or youth* or teenage* or kid or kids or baby or babies).ti,ab,kw

2. (antibiotic stewardship OR antimicrobial stewardship).ti,ab,kw

3. (PICU or Pediatric ICU or Pediatric Intensive Care Unit or Pediatric critical care).ti,ab,kw

4. 1 and 2 and 3

5. 4 not (review* or conference abstract* or letter* or note* or editorial*).pt.

6. Limit 5 to yr="2007-2024"
